# Supplementary figures and images for: An update on oral clinical courses among patients with severe acute respiratory syndrome coronavirus 2 (SARS-CoV-2) infection: A clinical follow-up (a prospective prevalent cohort) study
Source: PLoS One. 2022 Oct 21;17(10):e0275817. doi: 10.1371/journal.pone.0275817 (PMC9586351; doi:10.1371/journal.pone.0275817)

S1 Fig. WHO oral assessment form for adults and children


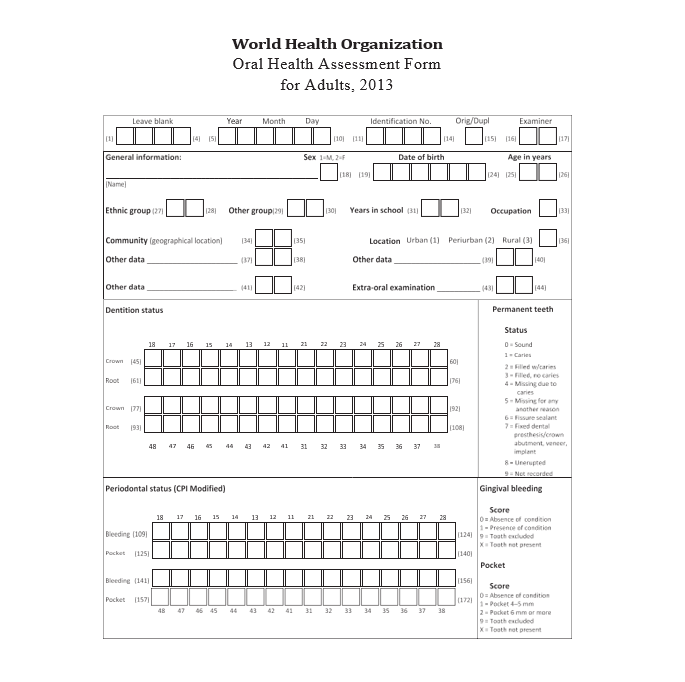


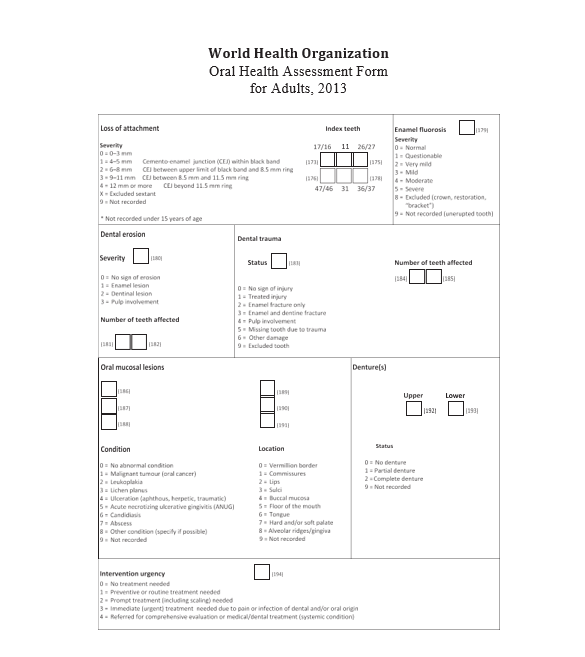


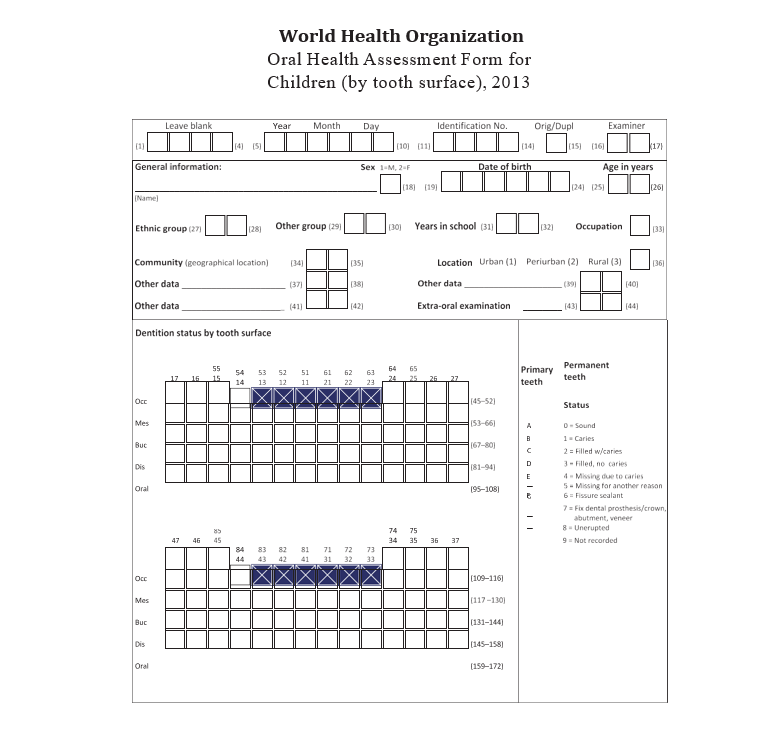


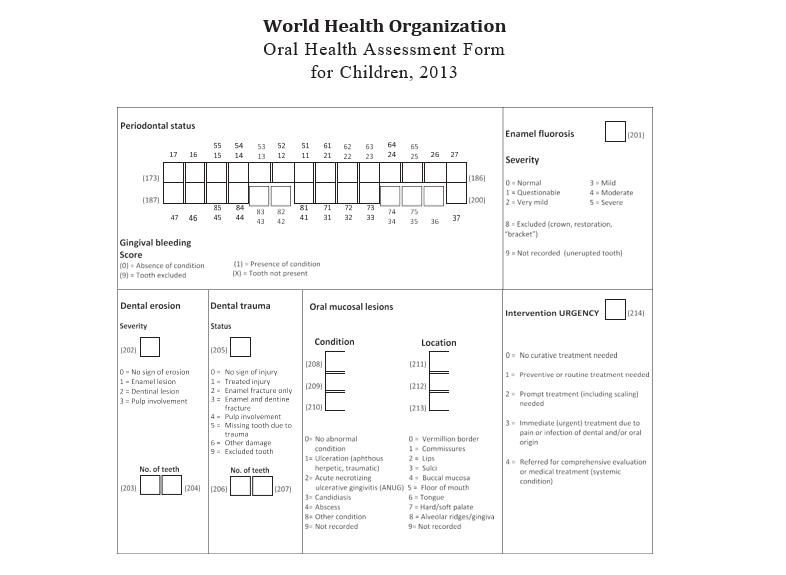

Supplement: S1 Fig — (DOCX) [file pone.0275817.s001.docx]

S2 Fig. The modified oral assessment form for covid-19 patients


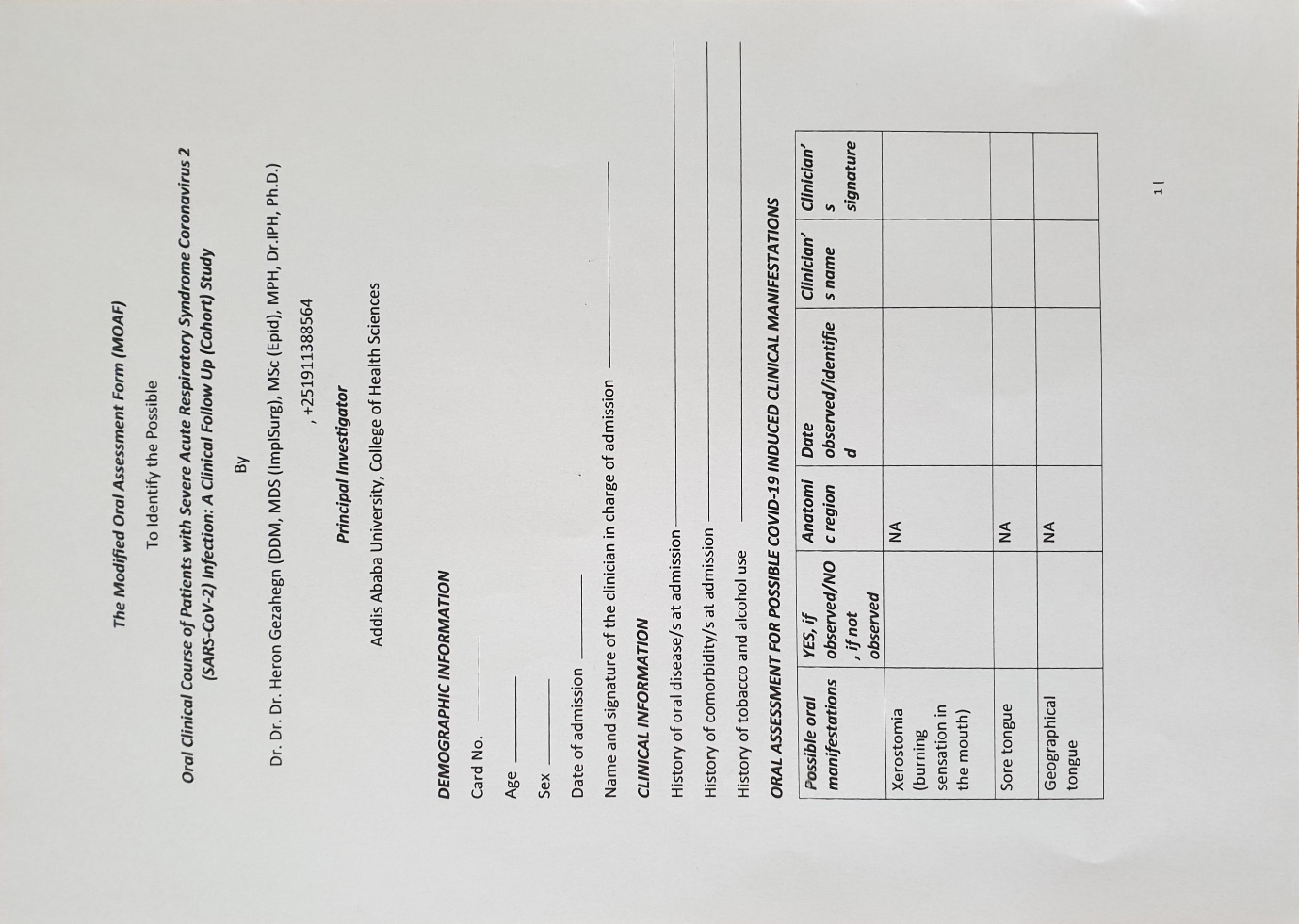


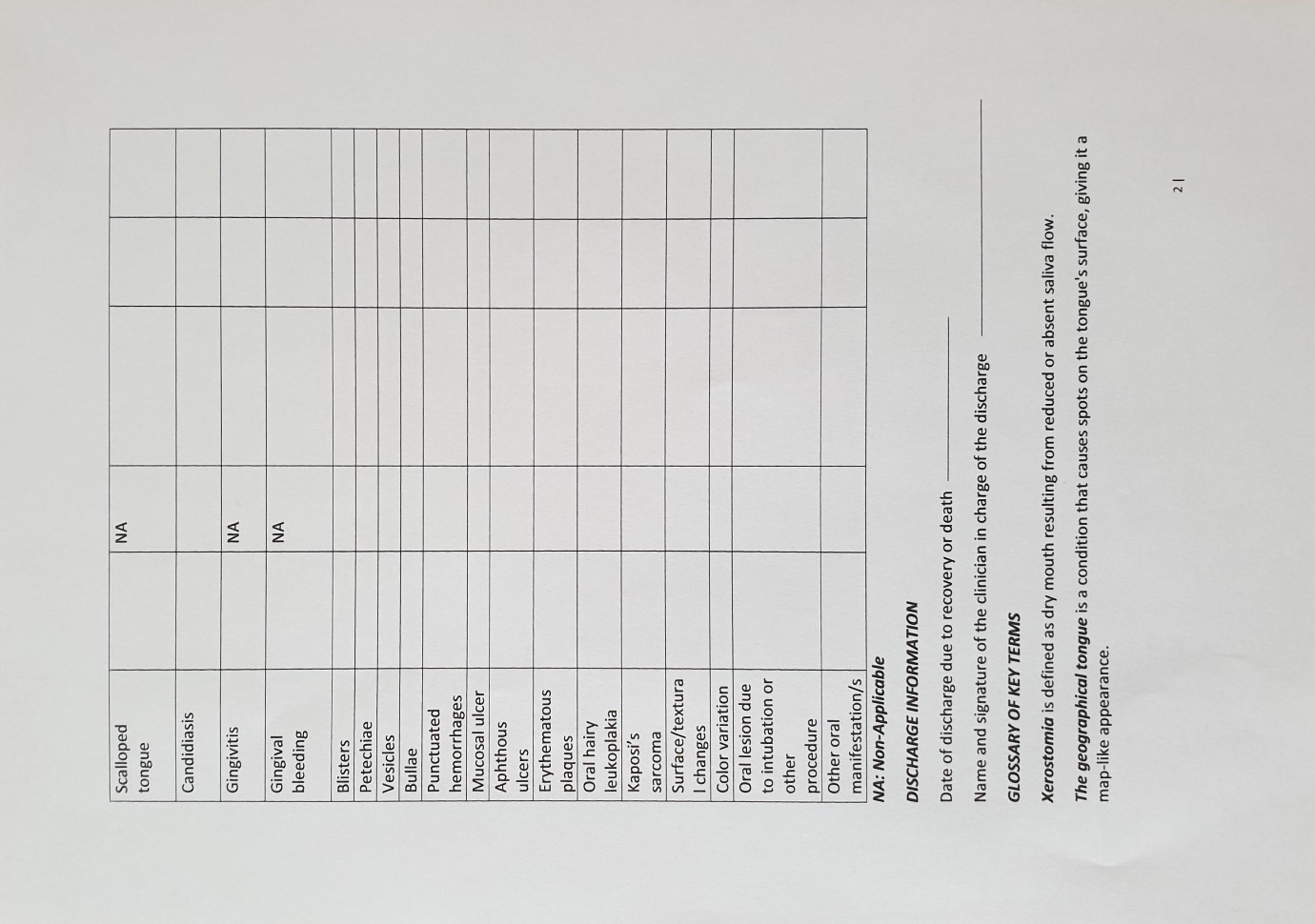


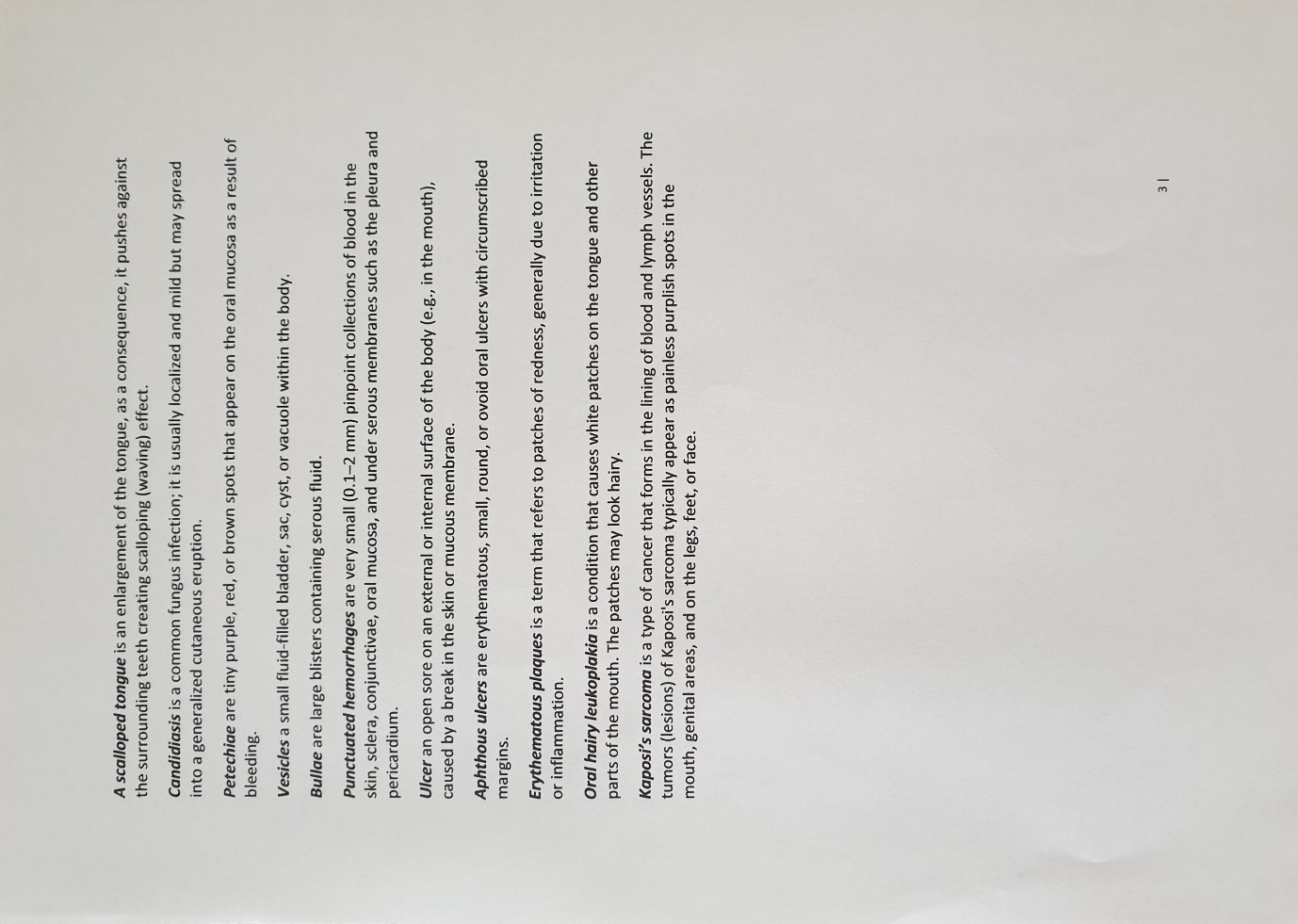

Supplement: S2 Fig — (DOCX) [file pone.0275817.s002.docx]
