# Supplementary material for: An update on oral clinical courses among patients with severe acute respiratory syndrome coronavirus 2 (SARS-CoV-2) infection: A clinical follow-up (a prospective prevalent cohort) study
Source: PLoS One. 2022 Oct 21;17(10):e0275817. doi: 10.1371/journal.pone.0275817 (PMC9586351; doi:10.1371/journal.pone.0275817)
Supplement: Attachment — Submitted filename: Response to Reviewers.docx [file pone.0275817.s003.docx]

**Response to reviewers**

**PLOS ONE**

**Title**: **An update on oral clinical courses among patients with severe acute respiratory syndrome coronavirus 2 (SARS-CoV-2) infection: a clinical follow-up (a prospective prevalent cohort) study**

**Manuscript Number: PONE-D-22-19013**

*Dear Etsuro Ito,*

*I am delighted that my manuscript has been reviewed for publication by PLOS ONE. Thank you very much for your constructive feedback and helpful guidance.*

*I have taken the reviewer and editor’s comments very seriously and have invested a great amount of work to adequately address the concerns described in your decision email. I copied the reviewer and editor comments as presented in the email. For clarity, I bolded the comments as presented below. I responded to the editor’s and reviewer’s comments individually, indicating exactly how I addressed each concern or problem and describing the changes made. Two versions of the revised manuscript: one with highlights denoting where the text has been changed; the other a clean version are uploaded as the manuscript file. Contents removed and added to the highlighted manuscript are colored in red and green, respectively. I hope that you approve the taken steps and look forward to hearing from you soon.*

*The author.*

***Comment of editor***

***Please clarify the inclusion criteria.***

*Thank you very much for your suggestion. I fully agree with your comment to clarify the inclusion criteria. Accordingly, I brought the inclusion criteria to your attention in the “study location and population” section of the revised manuscript.*

***The first comment of Reviewer #1***

***Line 55: As a recommendation, the authors can comment on the most recent influenza pandemic of 2009, which occurred 10 years before the new SARS-Cov-2 pandemic. In the introduction, the pandemic of more than 100 years ago (1918) is very well cited.***

*Thank you very much for your valuable and very helpful comment. I have included a brief note about the 2009 H1N1 influenza virus in the introduction section of the revised manuscript based on your comment.*

***The second comment of Reviewer #1***

***Line 83: At the end of the line, modify the citation (Xu et al., 2020), since the references are only numerical.***

*Thank you very much for making me aware of this point. I adapted the intext citation to numerical using Zotero.*

***The third comment of Reviewer #1***

***Line 107: Do not describe the objective as it is written in this section, because it is very well justified in line 112.***

*Thank you very much for this useful suggestion. The recommended modification has been made based on your comment.*

***The fourth comment of Reviewer #1***

***Line 127: It is essential to first propose the inclusion criteria. Identify the age ranges that can be included. There is a calculation to obtain the sample of the study population, it was random, it was stratified. It is a prospective study; it is very important to know how these parameters were made. If these methodologies were not considered in the project, it can be justified by writing that they included 55 patients on a certain date. As an example: from February 1, 2022 to March 1, 2022 (on the date that was the study protocol), 55 patients (36 men and 19 women) were included, in the format the age is asked, as a recommendation, they can calculate the ages of the men and women who participated. In the inclusion criteria, indicate what ages could be included in the study protocol.***

*Thank you very much for highlighting these important aspects. Each of these recommendations is addressed in the revised manuscript under the* *“study location and population” section.*

***The fifth comment of Reviewer #1***

***Line 146: It is not mentioned which variables were collected and how they would be measured, it is only limited to an Excel sheet (an attached document on the study parameters is presented), the study variables must be specified and how they will be measured in the section of data analysis. This section must include the different variables that would be studied and how they will be measured, as an example the variables that are reported in table 1 of line 180 and in table 2 of line 197.***

*Thank you very much for your constructive suggestion. I fully agree with your comment to specify the study variables and describe the method by which they are measured in the data analysis section. Accordingly, the recommended changes are made in the data analysis. Now the data analysis section elaborated further in detail.*

***The sixth comment of Reviewer #1***

***Line 148: The objective does not mention which variables will be collected and how they will be measured, place it in the section of line 146.***

*Thank you for bringing this important point to my attention. The issue is addressed in the* *“study design” and “data analysis” sections of the revised manuscript.*

***The seventh comment of Reviewer #1***

***Line 162: The results are well written and clearly presented, but it would make it much easier to set the inclusion criteria, which variables would be reported and how these variables would be measured.***

*Once again, thank you very much for this constructive suggestion. The recommended change has been made in the “study design”, “study location and population”, and “data analysis” sections of the revised manuscript based on this helpful comment.*

***The eighth comment of Reviewer #1***

***Line 286: The greatest strength of the article is the conclusion.***

*I would like to thank you very much for this positive and courageous feedback.*

*Dear Reviewer,*

*Thank you very much for your detailed revision of my manuscript. I also appreciate your insightful comments on all aspects of the manuscript. I made big efforts to adjust carefully every issue point-by-point and improve the manuscript quality as a scientific publication.*

*Once again,* *thank you very much for your most constructive and helpful comments. I appreciate the opportunity to revise my manuscript.*

*The author.*
